# Case Report: Reduced lactate response despite maintained performance: a case study of a world-class Paralympic sprinter undergoing pre-games hypoxic training

**DOI:** 10.3389/fspor.2026.1910721

**Published:** 2026-09-03

**Authors:** Kosuke Taniguchi, Kentaro Horiuchi, Naoki Wada, Yoko Mizuno, Tetsunari Nishiyama, Naoki Kikuchi, Masaaki Sugita

**Affiliations:** Faculty of Sport Science, Nippon Sport Science University, Tokyo, Japan

**Keywords:** 400-m sprint, blood lactate, case study, hypoxic training, para-athletics, Paralympic athlete, repeated-sprint training

## Abstract

This case study describes the physiological adaptations and performance outcomes of a high-intensity interval training in hypoxia (HITH) intervention in a world-class female Paralympic sprinter during her final preparation for the Paris 2024 Paralympic Games. The participant was a Tier 5, T47 national record-holder in the 400 m. Over an approximately 13-week (≈3-month) period leading up to the Games, she performed HITH on a cycle ergometer, generally twice weekly, in a normobaric hypoxic chamber (FiO_2_ 14.5%, ∼3,000 m). Performance was assessed via periodic anaerobic power tests and official 400-m race times, and 3-min post-set blood lactate concentration was measured after each HITH session. Throughout the intervention, no clear improvement was observed in maximal or mean power output during laboratory tests, and the athlete's 400-m time at the Paralympic Games was consistent with her 2023 season best, indicating performance maintenance. During the intervention, 3-min post-set blood lactate during repeated-sprint sessions conducted within a broadly consistent framework showed an overall downward trend (Set 1: ∼21 to ∼18 mmol·L⁻¹) while mean and peak power were maintained or slightly increased. The HITH intervention was not associated with enhanced maximal power or a faster 400-m time; the reduced blood lactate response is interpreted cautiously and, because potential mechanisms were not directly measured, is offered as a hypothesis of altered lactate kinetics rather than a demonstrated adaptation.

## 1 Introduction

The 400-meter sprint (400-m) is a demanding event that relies heavily on both the anaerobic and aerobic energy systems to sustain high-speed running (1). To enhance these capacities, various training modalities have been developed, among which high-intensity interval training in hypoxia (HITH) has gained attention as a potent method for improving sea-level sprint and endurance performance (2).

Kim and Hong (3), who investigated changes in the event composition (disciplines) of Paralympic track and field from the Athens 2004 to the Tokyo 2020 Games, as well as trends and differences in performance among top finishers (podium: No. 1–3) and finalists (No. 1–8) by classification and event, aimed to provide fundamental data that could contribute to improving athletic performance, competition management, and training in Paralympic athletics. Their findings indicated an overall trend of performance improvement across successive Games, which likely reflects the enhancement of the competitive level through advancements in training methods, equipment technology, and the expansion of the athlete pool. In light of these developments, the authors suggested that adopting more scientific approaches is essential for further improving performance in Paralympic track and field.

While the benefits of HITH are well-documented in able-bodied athletes, there is a paucity of research on its application to elite para-athletes, particularly during the crucial final preparation phase for a major international competition like the Paralympic Games. Case studies on world-class (Tier 5) athletes offer invaluable, real-world insights into individualized training responses that are often lost in group-average designs (4).

Upper-body movement, particularly arm swing, influences running biomechanics, and active arm swing provides metabolic and biomechanical benefits (5). Because upper-limb deficiency affects running mechanics and restricted arm swing alters metabolic responses (5, 6), we reasoned that athletes with upper-limb deficiency might experience greater velocity decline in the latter stages of a 400-m (a rationale rather than an established characteristic of this population). Accordingly, training strategies that target metabolic stress, such as hypoxic training, may be warranted. A cycle-ergometer HITH protocol was selected to deliver controlled, repeatable, and symmetric all-out efforts with quantifiable external power in a hypoxic chamber while limiting running-specific mechanical load; the athlete's T47 upper-limb deficiency further motivated a focus on metabolic (speed-endurance) capacity and on internal-load monitoring alongside external performance.

This study presents the case of a Paralympic athlete, a T47 national record holder and Paralympic medalist in the 400-m. We detail a proactive, data-driven support program involving a HITH intervention implemented in the final months leading up to the 2024 Paris Paralympics. The purpose of this case study is to describe the implementation and physiological responses to this intervention and to evaluate its impact on the performance of a world-class female para-athlete.

## 2 Materials and methods

### 2.1 Participant

The participant in this case study was a world-class female track and field para-athlete specializing in the 400-m sprint (Paralympic classification: T47) with a congenital right forearm deficiency (absence of the limb distal to the elbow). She was 29 years old, with a body height of 158 cm and a body weight ranging from 46.0 to 48.4 kg. She is a national record holder (58.45 s, set on 24 April 2021) and international medalist, corresponding to a “Tier 5” athlete according to the classification proposed by McKay et al. (7). The athlete provided written informed consent for participation in the study, data collection, and publication. The study protocol was approved by the Ethics Committee of Nippon Sport Science University (Approval No. 023-H079) and was conducted in accordance with the Declaration of Helsinki.

### 2.2 Study design

A single-subject, longitudinal design was employed, following a proactive model of ongoing support integrated into the athlete's training schedule.

### 2.3 Intervention

The intervention took place from May to August 2024. A total of sixteen HITH sessions were completed over approximately 13 weeks; sessions were generally scheduled twice weekly, with interruptions for competitions and training camps. The athlete performed HITH in a normobaric hypoxic chamber (YHS-C10A, YKS, Nara, Japan) set to a simulated altitude of approximately 3,000 m (FiO_2_ 14.5%). A typical session consisted of two sets of six 25-s all-out sprints on a cycle ergometer (Fujin-Raijin, O.C. Labo, Tokyo, Japan), with 50 s (reduced to 40 s in the final two sessions, 5 and 10 August) of recovery between sprints and 20 min between sets; on one occasion only a single set was performed (3 June, Phase 2).

The HITH protocol employed in this study was designed to enhance both sea-level sprint and endurance performance. The work-to-rest ratio (1:2) commonly used in Repeated-Sprint Training in Hypoxia (RSH) was adopted (8). While conventional Sprint Interval Training in Hypoxia (SIH) or other sprint interval training (SIT) protocols that primarily aim to enhance glycolytic enzyme activity (e.g., phosphofructokinase; PFK) typically use a resistance of 0.075 kp·kg⁻¹ of body mass, in the present study a lower resistance of 0.03–0.025 kp·kg⁻¹ was applied (9). The training protocol adopted in this study was selected to improve velocity decline in the latter stages of a 400-m, based on discussions with the athlete and coach. Table 1 presents the schedule of the periodic tests and hypoxic training sessions.

**Table 1:** The schedule of the periodic tests and hypoxic training sessions. HITH, high-intensity interval training in hypoxia; 10-MAT, 10-s maximal anaerobic power test; 40-MAT, 40-s maximal anaerobic power test. ○ indicates a session/test performed on that date.

Date	HITH	10-MAT	40-MAT	Jump
4/20		○	○	
5/8	○	○		
5/10	○	○		○
5/29	○	○		
5/31	○	○		○
6/3	○	○		
6/21	○	○	○	
6/24	○	○		○
6/28	○	○		○
7/1	○	○		
7/18	○	○	○	○
7/22	○	○		
7/25	○	○		○
7/29	○	○		
8/1	○	○		○
8/5	○	○		
8/10	○	○		○

### 2.4 Data collection

#### 2.4.1 Performance assessment

Periodic tests included a 10-s maximal anaerobic power test (10-MAT) and a 40-s maximal anaerobic power test (40-MAT). The 10-MAT and 40-MAT were performed on a cycle ergometer (Fujin-Raijin, O.C. Labo, Tokyo, Japan) using a resistance of 1.0 kp for the 10-MAT and 0.075 kp·kg⁻¹ of body mass for the 40-MAT. The 10-MAT was performed before each HITH session and on 20 April (*n* = 17 in total). The 40-MAT was performed three times in total: once before the intervention (20 April) and twice during it (21 June and 18 July). On-track performance was evaluated by comparing the official 400-m time at the Paris Paralympics with the athlete's 2023 season best (59.09 s). Because the national record (58.45 s) was set in April 2021 and had not since been improved—one motivation for adopting hypoxic training—the most recent full-season best (2023: 59.09 s) was used as the primary comparator. The final HITH session (10 August) was performed approximately three weeks before the Paralympic 400-m race (31 August); HITH was then discontinued for competition preparation, including an off-site pre-competition camp.

#### 2.4.2 Physiological monitoring

Capillary fingertip blood samples were taken 3 min after the end of each set (i.e., 3 min after Set 1 and 3 min after Set 2) to determine the 3-min post-set blood lactate concentration (Lactate Pro 2, Arkray, Kyoto, Japan; measurable range 0.5–25.0 mmol·L⁻¹). The Set-1 sample was obtained at the start of the 20-min inter-set recovery, and no further sample was taken until 3 min after Set 2. On four occasions the reading exceeded the analyzer's upper limit (“Hi”: Set 1 on 3 June, 21 June and 1 July, and Set 2 on 1 July) and was recorded as the upper-limit value (25.0 mmol·L⁻¹); these right-censored values are flagged in Supplementary Table S4, and their potential influence on the temporal trend is noted in the Limitations. The 3-min post-exercise sampling time was chosen to approximate the time-to-peak of blood lactate following maximal efforts, and the same sampling procedure was applied consistently across all sessions. The session sequence was kept consistent across sessions (approximately 10 min of self-directed warm-up, followed by the countermovement jump measurement, the 1-kp 10-MAT, and then the HITH session); the warm-up consisted of self-directed exercise whose precise content and intensity were not rigidly standardized. Sessions were generally performed in the afternoon (∼15:00–17:00) but, owing to the athlete's competitive schedule, were on some occasions performed in the late morning (∼11:00–12:30); time of day was therefore recorded but not standardized. Nutritional intake, prior-day training load, and recovery status were not monitored or recorded. These uncontrolled factors may have contributed to session-to-session variability in the blood lactate response and are acknowledged as a limitation.

#### 2.4.3 Pedaling performance

Daily training data were collected by assessing both peak and mean power output during each HITH session.

#### 2.4.4 Jump performance

Countermovement jump (CMJ) height was monitored regularly throughout the intervention period. CMJ height was measured with a Multi Jump Tester II (Q'sfix, Tokyo, Japan). After a self-selected warm-up, the athlete performed two countermovement jumps with arm swing and the better of the two was retained; the recovery interval between trials was not recorded and no separate familiarisation was undertaken. Given the participant's congenital right forearm deficiency, jumps were performed with the available arm swing.

#### 2.4.5 Athlete's training

Information on the athlete's training protocol was collected through interviews with the coach.

### 2.5 Statistical analyses

**Figure 1:**
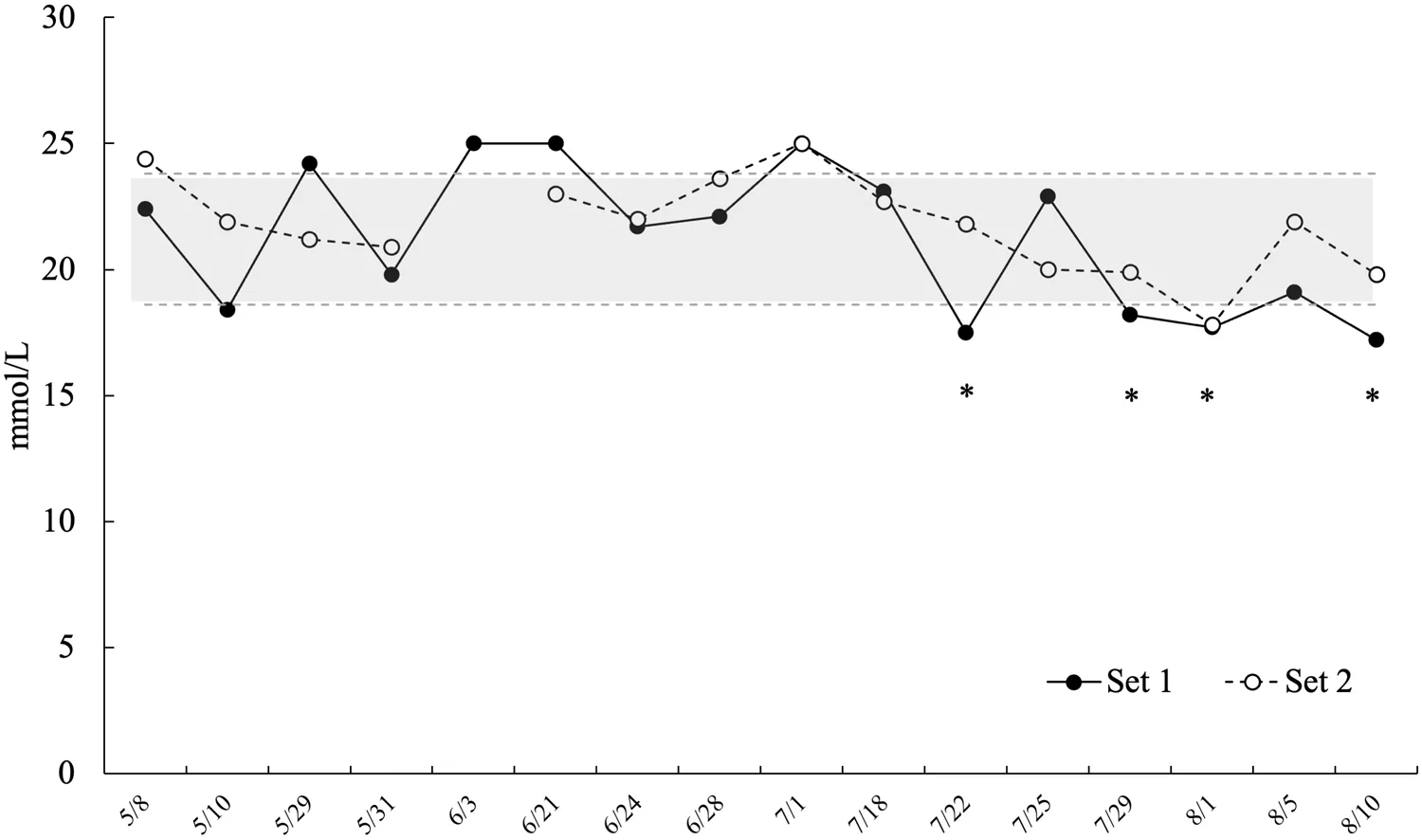
3-min post-set blood lactate concentration during high-intensity interval training in hypoxia (HITH) sessions. The shaded band denotes the baseline variability band (baseline mean ± one SD of the first four Set-1 observations; 18.6–23.8 mmol·L⁻¹); asterisks mark later Set-1 sessions falling below the band. Values that reached the analyzer's upper limit (25.0 mmol·L⁻¹; “Hi”) are plotted at 25.0 and were right-censored (see Supplementary Table S4).

**Figure 2:**
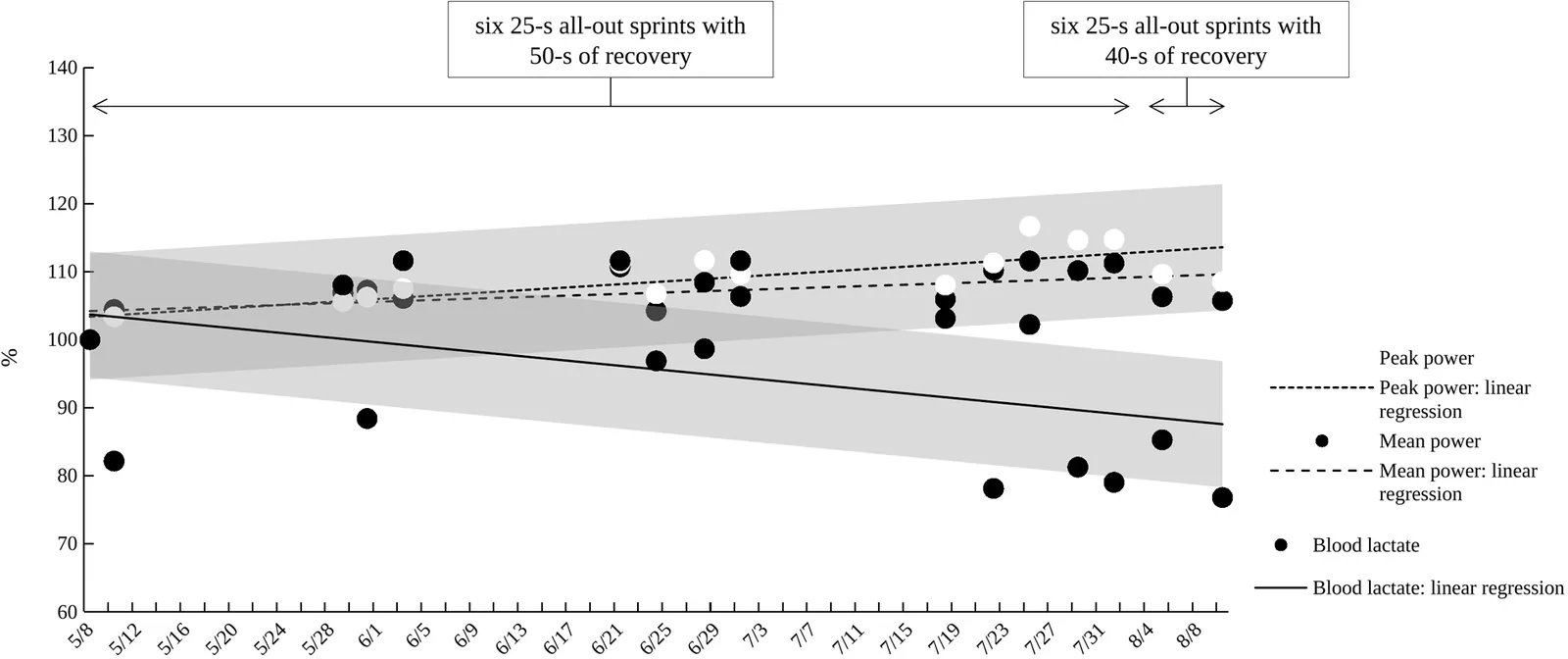
Relative changes in blood lactate, mean power, and peak power across the intervention period for Set 1. Values are expressed relative to the first session (=100%). The solid and dashed regression lines are simple ordinary-least-squares fits computed across the whole intervention for each variable, with shaded 95% confidence intervals; they are not the phase-stratified, power-adjusted models reported in Supplementary Table S2.

Data were calculated and presented as mean (SD) and were analyzed using Microsoft Excel (Microsoft, Redmond, WA, USA). To examine temporal changes in blood lactate after adjustment for external power output, multiple linear regression analyses were conducted. Separate models were constructed for mean power and peak power, with blood lactate as the dependent variable and time (days from the start of the intervention) and power output entered as independent variables. Based on the athlete's training periodization (Supplementary Table S1), phase-stratified linear regressions were fitted for Phase 1 (8–31 May; *n* = 4), Phase 2 (3 June–29 July; *n* = 9), and Phase 3 (1–10 August; *n* = 3). To visualise session-to-session variability, a baseline variability band was defined for Set 1 (Figure 1) as the mean ± one standard deviation (SD) of the first four Set-1 observations (8–31 May); this band represents baseline within-athlete variability and is not a smallest-worthwhile-change estimate. Regression analyses were performed using SPSS Statistics version 29 (IBM Corp., Armonk, NY, USA). Statistical significance was set at *p* < 0.05. Given the single-case design and the small per-phase sample, the regression models were treated as exploratory; interpretation therefore emphasized the direction and consistency of effects and descriptive indicators rather than statistical significance alone. For Figure 2, blood lactate, mean power and peak power (Set 1) were each expressed relative to the value at the first session (set to 100%); the regression lines and 95% confidence intervals in Figure 2 are simple ordinary-least-squares fits across the whole intervention for each variable, and are distinct from the phase-stratified, power-adjusted models in Supplementary Table S2.

## 3 Results

### 3.1 Athlete's training

An overview of the athlete's track and strength training performed during the intervention period is presented in Supplementary Table S1. The athlete's training program was structured in three phases across the competitive season. From April to June, the primary focus was on improving 100-m sprint speed while competing in major events, with the aim of enhancing the first half of the 400-m race. From June to July, training transitioned from short-distance speed development to longer-distance work specific to the 400 m, following a brief reduction in training load. This phase included training camps and was characterized by high training intensity. In the final phase, from August to the pre-competition period, training was directed toward integrating previous adaptations and optimizing performance for the target competition. The program incorporated a combination of speed and endurance training, including interval running over 200 m and occasional longer-distance runs (e.g., 500 m), as well as rhythm-based pacing and speed control exercises. In addition, resistance training and resisted sprint exercises were performed alongside regular track training.

### 3.2 Competition performance

Table 2 presents the athlete's 400-m competition performance during the 2024 season. The athlete's 400-m time at the Paralympic Games was comparable to her 2023 season best (59.09 s). Performance was therefore maintained during the intervention period. Because championship performance is influenced by many factors (e.g., tapering, travel, psychological state, race context, and environmental conditions), this maintenance is reported as an observation and is not attributed to the HITH intervention.

**Table 2:** The athlete's 400-m competition performance during the 2024 season.

Date	Competition	400-m time (s)
9 Jun 2024	National Championships	60.19
22 Jun 2024	Time trial meet	61.00
27 Jul 2024	Time trial meet	59.09
9 Aug 2024	Time trial meet	59.07
31 Aug 2024	Paris Paralympics	59.13
2023 season best	—	59.09

### 3.3 Performance assessment

No clear or systematic improvement was observed in peak or mean power during the anaerobic capacity tests throughout the intervention period (Supplementary Figure S1).

### 3.4 Physiological response

Despite the stability in performance metrics, an overall downward trend in 3-min post-set blood lactate concentration was observed (Figure 1). The athlete was able to complete a broadly consistent training workload with a substantially lower lactate response in the final weeks of the intervention compared with the initial weeks. As illustrated in Figure 1, blood lactate concentrations for Set 1 in the later sessions fell below the lower bound of the baseline variability band; this indicates values below the range of baseline within-athlete variability. Descriptively, mean 3-min post-set blood lactate (Set 1) decreased from 21.2 mmol·L⁻¹ during the baseline phase (first four sessions) to 18.0 mmol·L⁻¹ in August (−15%), while over the same period mean power increased by 3.0% (201.9–208.0 W) and peak power by 6.9% (239.9–256.6 W). The baseline value was the mean of the first four sessions (8, 10, 29, and 31 May) and the August value the mean of three sessions (1, 5, and 10 August); all comparisons used Set 1. Two of the three August sessions (5 and 10 August) used a shortened inter-sprint recovery (40 s vs. the usual 50 s; sprint duration unchanged at 25 s) and were included in the August mean; because the metabolic effects of shortening recovery during repeated maximal exercise are complex, this modification is treated as a source of uncertainty rather than as evidence supporting the lactate finding. The single-set session (3 June) occurred within Phase 2 and was not included in the August comparison.

### 3.5 Pedaling performance

No clear improvement was observed in peak power output throughout the intervention period. Mean power output showed an increasing trend from the early to mid-phase of the intervention (Supplementary Figure S2).

### 3.6 Jump performance

CMJ height showed a gradual declining trend throughout the intervention period (Supplementary Figure S3). Linear regression indicated a decline of approximately 0.24 cm·week⁻¹ (∼0.6% per week; from 40.1 to 36.4 cm overall, −9%; r = −0.44, *p* = 0.28). Because these are a single time series with an essentially constant weekly hypoxic exposure, the trend is reported descriptively and was not modelled as a dose–response.

### 3.7 Temporal trends in blood lactate

Scatter plots illustrating the relative changes in blood lactate, mean power, and peak power across the intervention period for Set 1 are presented in Figure 2. Visual inspection suggested an overall downward trend in blood lactate over time, whereas peak and mean power remained relatively stable. Results of the phase-stratified linear regression analyses are shown in Supplementary Table S2; models were fitted separately for Phase 1 (8–31 May, *n* = 4), Phase 2 (3 June–29 July, *n* = 9), and Phase 3 (1–10 August, *n* = 3). In Phase 1, blood lactate remained relatively stable. During Phase 2, although the temporal trends did not reach statistical significance (*p* = 0.066–0.099), consistent negative slopes for time were observed across all models (B = −0.102 to −0.127). In Phase 3, the limited number of sessions (*n* = 3) precluded formal inferential statistics, but descriptive data suggested the maintenance of a lowered lactate response.

## 4 Discussion

The primary finding of this case study is that while an approximately 13-week HITH intervention did not improve maximal power or 400-m race time, it was accompanied by a notable physiological change, evidenced by a blunted blood lactate response to repeated high-intensity exercise. We interpret this cautiously as being consistent with a reduced lactate response and/or altered lactate kinetics; however, because this is a single-case design conducted alongside concurrent training, a causal link between the intervention and the maintenance of her performance cannot be established.

Importantly, phase-stratified regression analyses adjusting for both mean and peak power output showed negative temporal slopes in blood lactate responses particularly during Phase 2, indicating that the trend was not clearly associated with reductions in external mechanical output. However, the very small per-phase samples preclude firm inference; the consistent direction of the coefficients is noted descriptively and should not be interpreted as confirmatory evidence of an independent temporal effect.

For a top-tier athlete, the ability to consistently reproduce peak performance at major championships is as important as achieving new personal bests. The observed outcome—performance maintenance accompanied by a reduced lactate response—is an interesting observation, but the present single-case design cannot establish that it reflects successful conditioning.

The fact that lactate responses declined despite comparable power production may further support the possibility of physiological adaptations related to lactate kinetics or energy metabolism. These changes may reflect altered lactate kinetics, such as increased lactate clearance or utilization, or a greater contribution from aerobic metabolism during repeated sprints, thereby reducing reliance on anaerobic glycolysis, although this study cannot directly determine the underlying mechanisms (10). Importantly, these candidate mechanisms were not directly measured—no assessment of oxygen uptake, lactate clearance rate, muscle oxygenation, ventilatory responses, pH, or bicarbonate was performed—and they are therefore presented only as hypotheses rather than confirmed adaptations.

### 4.1 Comparison with able-bodied hypoxic training

Our observation contrasts with the able-bodied repeated-sprint-training-in-hypoxia (RSH) literature. Some RSH and sprint-interval-hypoxia studies have reported increased maximal blood lactate [Trincat et al., 2017 (11)] or enhanced glycolytic markers [e.g., phosphofructokinase activity; Puype et al., 2013 (9)], and improved repeated-sprint ability with greater muscle perfusion [Faiss et al., 2013 (12)], whereas the present athlete showed lower post-set lactate at a maintained workload. This divergence is consistent with the deliberately low-resistance, cadence-oriented protocol combined with concurrent 400-m endurance training, which together may bias the stimulus toward an oxidative/clearance-oriented rather than a glycolytic adaptation. A literature search (PubMed; searched July 2026; terms combining “hypoxia/altitude”, “repeated-sprint/sprint-interval training”, and “Paralympic/para-athlete/amputee”) identified no prior hypoxic-training intervention study in para-sprint athletes; the available evidence is confined to able-bodied cohorts (Supplementary Table S3).

### 4.2 Upper-limb deficiency and lactate kinetics in the T47 sprinter

The participant's congenital forearm deficiency (T47) restricts arm swing, which is not a trivial asymmetry: active arm swing reduces the metabolic cost of running, and its restriction alters whole-body energetics (5, 6). A reduced or asymmetric arm swing may accentuate the velocity decline in the final 100–150 m of a 400-m race, providing the rationale for targeting metabolic (speed-endurance) capacity. Notably, an upper-body RSH study in cross-country skiers reported enhanced repeated-sprint ability and upper-limb perfusion [Faiss et al., 2015 (13)]; however, that evidence derives from able-bodied athletes with intact limbs. Whether the blunted lactate response observed here represents a disability-specific adaptive signature or a general hypoxic/endurance adaptation cannot be resolved from a single case and is presented as an open question for future controlled work.

The sprint duration was set to 25 s, based on pilot data indicating that this duration corresponds to the time before the pedal cadence declines to below 80% of the peak value during a 40-s maximal anaerobic power test. This adjustment was made to target both sprint and endurance performance by imposing not only metabolic but also additional neuromuscular stress; the protocol was intentionally designed to maintain high pedaling cadence and thereby elicit substantial neuromuscular stress.

In the present case, the observed reduction in blood lactate response during the HITH intervention period may have been influenced by the concurrent track training program. While the primary focus from April to June was on enhancing 100-m sprint speed, the training from June to July transitioned toward 400-m-specific endurance, incorporating higher-intensity, longer-distance work such as 200-m intervals and occasional 500-m runs. The strategic integration of this high-intensity, specific training phase with the HITH sessions may have coincided with the maintenance of speed endurance and may have contributed to the observed reduction in lactate response during the HITH sessions. While the phase-stratified regression models did not reach statistical significance, likely due to the limited sample size inherent in a single-case design, they provide descriptive support for this phase-specific nature of the adaptation. Specifically, a consistent downward trajectory in the blood lactate response appeared to begin during Phase 2, which precisely coincides with the transition to 400-m specific endurance training, suggesting a potential targeted metabolic adaptation.

In the context of the 400-m event, fatigue accumulation influences the latter stages of the race. A lower blood lactate response at a given workload could, in principle, be consistent with improved metabolic tolerance, which might relate to speed endurance and the rate of deceleration in the final 100–150 m; however, the functional significance of this pattern remains uncertain because late-race split performance and lactate kinetics were not directly assessed. We therefore refrain from concluding that the intervention improved the athlete's ability to sustain speed.

During the intervention period, a downward trend was observed in CMJ performance (Supplementary Figure S3). This suggests that the intensity or volume of strength and power training included in the program might have been insufficient to maintain neuromuscular performance. Previous studies have reported that concurrent endurance and strength training can induce an interference effect, potentially impairing gains in muscle strength and power performance (14, 15). Furthermore, endurance training under hypoxic conditions has been shown to markedly activate skeletal muscle AMPK signaling and increase oxidative stress (16), potentially amplifying the inhibitory influence on muscle strength adaptation. Therefore, future training interventions should consider incorporating a more balanced approach that includes sufficient strength- or power-oriented stimuli to counteract these potential negative effects. The reasons for the CMJ decline in this single athlete cannot be established; the mechanisms above (concurrent-training interference, AMPK signalling) are offered only as possible explanations.

This case study underscores a methodological point: performance tests of maximal capacity (e.g., 40-MAT) may not capture all training-related changes. To fully understand an athlete's response to training, it is useful to combine measures of external output (power, time) with measures of internal physiological response (lactate, heart rate). For coaches and practitioners, this means that even if performance metrics plateau, changes in internal physiological markers may still be occurring and warrant monitoring.

### 4.3 Limitations

A limitation of this intervention period is that the measurement conditions were not fully standardized across assessments. Variations in sport-specific training or strength training may have prevented the use of consistent testing conditions. This study involved a single elite athlete without a control condition. Therefore, the observed changes in lactate response cannot be exclusively attributed to the HITH intervention. Furthermore, detailed daily external load metrics (e.g., total running distance, session RPE) from the track training sessions were not continuously quantified. Consequently, we were unable to perform lagged regression analyses to statistically control for the potential effects of carryover fatigue on blood lactate responses. Additional limitations should be noted. The findings derive from a single athlete and have limited generalizability. The proposed mechanisms underlying the reduced lactate response were not directly examined. No matched pre-HITH lactate baseline was available, because capillary lactate sampling began with the first HITH session. Protocol modifications were introduced during the intervention (reduced inter-sprint recovery in the final two sessions and one single-set session), which may have influenced the lactate response. Finally, the low-resistance, high-cadence cycling protocol (0.025–0.03 kp·kg⁻¹) has limited biomechanical transfer to the running gait of an athlete with congenital upper-limb deficiency; future trials could contrast higher-resistance hypoxic sprint cycling or running-based modalities to better replicate race-specific neuromuscular loading in T47 athletes. Finally, apart from a consistent session sequence, the warm-up was self-directed and nutritional intake, time of day, prior-day training load, and recovery status were not standardized or recorded, and two of the three August sessions used a shortened inter-sprint recovery; these factors may have influenced the session-to-session and baseline-versus-August blood lactate comparisons. In addition, four early-to-mid-intervention values reached the analyzer's upper limit and were entered as 25.0 mmol·L⁻¹ (right-censored); because they occurred before the August period, they did not affect the baseline-versus-August descriptive comparison but may have attenuated the estimated within-intervention temporal slope.

### 4.4 Practical applications

This case demonstrates that HITH may be accompanied by a reduced lactate response during repeated high-intensity exercise, even if it does not increase maximal power output. Coaches should consider monitoring internal physiological markers (e.g., blood lactate) alongside external performance data to obtain a more complete picture of training adaptation. A blunted lactate response to a given workload should be interpreted alongside external power, perceived exertion, training context, recovery status, and competition performance, as it may also reflect factors such as substrate availability, accumulated fatigue, or pacing changes rather than adaptation alone.

For practitioners, this case also illustrates a trade-off: the HITH block was associated with a lower post-session lactate at a maintained workload but coincided with a concurrent decline in vertical-jump performance (∼9% decrease in CMJ), the functional consequences of which were not directly assessed. We therefore suggest pairing metabolic and neuromuscular monitoring and protecting neuromuscular qualities when integrating HITH into pre-Paralympic tapering. A balanced weekly microcycle for a 400-m sprinter with congenital upper-limb deficiency might reduce HITH from twice to once weekly during strength-emphasis blocks, add a low-intensity hypoxic active-recovery session, and integrate upper-limb and vertical-power plyometrics to offset CMJ decline, with weekly paired monitoring of CMJ and blood lactate. This is presented as a practice-informed proposal to be tested rather than a validated prescription.

## 5 Athlete perspective

From my perspective as the athlete, the final months before the Paris 2024 Games were physically demanding, but the structured hypoxic sessions gave me confidence that I could hold my speed through the latter part of the 400 m. Although my maximal power and race time did not change, I felt that I recovered better between hard efforts and that the same sessions gradually became easier to complete as the weeks went on. Being able to see my own monitoring data—especially the changes in blood lactate—helped me trust the plan and stay motivated during a stressful pre-competition period. I hope that sharing my experience can support other para-athletes and coaches who are considering similar approaches.

## 6 Conclusion

In this world-class T47 sprinter, an approximately 13-week HITH programme conducted alongside sport-specific training was associated with an overall downward trend in 3-min post-set blood lactate during repeated-sprint sessions performed within a broadly consistent intervention framework (notwithstanding protocol modifications, including one single-set session and shortened inter-sprint recovery in two late sessions), while maximal power and 400-m performance were maintained. Causal attribution to HITH is not claimed; the reduced lactate response is offered as a monitoring insight that may be useful for para-sport practitioners, and its underlying mechanisms and disability-specific nature warrant future controlled investigation.

## Data Availability

The raw data supporting the conclusions of this article will be made available by the authors, without undue reservation.

## Supplementary material

SUPPLEMENTARY TABLE S1 Overview of the track and strength training program during the intervention period. The Weight 1 program emphasized upper-body strength and explosive-power exercises (dumbbell fly/press, lat pulldown, arm curl, bent-over front raise/side raise/rowing, high clean, sit-up, back high-power extension); the Weight 2 program centered on lower-body strength exercises (half squat, leg curl, leg raise, hip lift, leg lunge, split squat) and core training (training loads ≈60%–85% 1RM).

SUPPLEMENTARY TABLE S2 Temporal changes in blood lactate after adjustment for external power output. Values are unstandardized regression coefficients (B) with 95% confidence intervals. Time represents days from the start of the intervention. Blood lactate was the dependent variable in all models. Phase 3 (1–10 August, n = 3) was not estimated because it provided zero residual degrees of freedom; descriptive results only.

SUPPLEMENTARY TABLE S3 Representative hypoxic repeated-/sprint-training studies and the present case. RSH, repeated-sprint training in hypoxia. Able-bodied RSH/SIH studies have reported increased maximal lactate, enhanced glycolytic markers, or improved repeated-sprint ability, whereas the present low-resistance HITH case showed reduced lactate at a maintained workload. A PubMed search (July 2026) identified no prior hypoxic-training intervention study in para-sprint athletes.

SUPPLEMENTARY TABLE S4 Session-by-session HITH details. Resistance was 0.025–0.03 kp·kg⁻¹ throughout; sprint duration was 25 s. Inter-sprint recovery was 50 s except in the final two sessions (5 and 10 August; 40 s). Power and blood lactate are shown for Set 1/Set 2. † indicates a value that reached the analyzer’s upper limit (“Hi”) and was recorded as 25.0 mmol·L⁻¹ (right-censored). — indicates no Set 2 (single-set session).

SUPPLEMENTARY FIGURE S1 Peak and mean power of the 40-s maximal anaerobic power test (40-MAT) (a) and the 10-s maximal anaerobic power test (10-MAT) (b).

SUPPLEMENTARY FIGURE S2 Peak power (a) and mean power (b) during high-intensity interval training in hypoxia (HITH) sessions. On one occasion only a single set was performed.

SUPPLEMENTARY FIGURE S3 Countermovement jump (CMJ) height across the intervention period.
