# Supplementary material for: Case Report: Reduced lactate response despite maintained performance: a case study of a world-class Paralympic sprinter undergoing pre-games hypoxic training
Source: Front Sports Act Living. 2026 Sep 3;8:1910721. doi: 10.3389/fspor.2026.1910721 (PMC13582420; doi:10.3389/fspor.2026.1910721)
Supplement: Supplementary file 1 [file Table1.docx]

**Supplementary Table S1 |** Overview of the track and strength training program during the intervention period. The Weight 1 program emphasized upper-body strength and explosive-power exercises (dumbbell fly/press, lat pulldown, arm curl, bent-over front raise/side raise/rowing, high clean, sit-up, back high-power extension); the Weight 2 program centered on lower-body strength exercises (half squat, leg curl, leg raise, hip lift, leg lunge, split squat) and core training (training loads ≈60–85% 1RM).

| **Phase** | **Period** | **Training focus** | **Track training (example)** | **Strength training** |
| --- | --- | --- | --- | --- |
| Phase 1 | Apr - Jun | Development of 100-m sprint speed and improvement of the first half of the 400-m race. | Short sprint training (60–120 m maximal sprints), resisted sprint drills, rhythm and speed-control exercises during the competition period. | Resistance training performed 1–2 sessions·week⁻¹ using two alternating programs (Weight 1 and Weight 2) consisting of 7–8 exercises. |
| Phase 2 | Jun - Jul | Transition to 400-m–specific endurance training following a brief reduction in training load. | 200–400 m interval runs, occasional longer runs (e.g., 500 m), high-intensity sessions during training camps. | Same resistance-training structure maintained (1–2 sessions·week⁻¹). |
| Phase 3 | Aug - Pre competition | Integration of training adaptations and competition preparation. | Race-pace running, pacing and rhythm-control drills, gradual reduction in training volume approaching competition. | Reduced-volume maintenance resistance training (1–2 sessions·week⁻¹). |
