# Supplementary material for: Case Report: Reduced lactate response despite maintained performance: a case study of a world-class Paralympic sprinter undergoing pre-games hypoxic training
Source: Front Sports Act Living. 2026 Sep 3;8:1910721. doi: 10.3389/fspor.2026.1910721 (PMC13582420; doi:10.3389/fspor.2026.1910721)
Supplement: Supplementary file 2 [file Table2.docx]

**Supplementary Table S2 |** Temporal changes in blood lactate after adjustment for external power output. Values are unstandardized regression coefficients (B) with 95% confidence intervals. Time represents days from the start of the intervention. Blood lactate was the dependent variable in all models.

| **Phase** | **Model** | **Set** | **Predictor** | **B (95% CI)** | **p** | **R²** | **Model p** |
| --- | --- | --- | --- | --- | --- | --- | --- |
| 1 | Mean Power | 1 | Mean Power | -0.516 (-7.928 to 6.897) | 0.539 | 0.478 | 0.722 |
|  |  |  | Time (day⁻¹) | 0.299 (-3.697 to 4.294) | 0.516 |  |  |
|  | Mean Power | 2 | Mean Power | -0.684 (-2.263 to 0.895) | 0.114 | 0.989 | 0.105 |
|  |  |  | Time (day⁻¹) | 0.094 (-0.399 to 0.588) | 0.248 |  |  |
|  | Peak Power | 1 | Peak Power | -0.623 (-8.112 to 6.865) | 0.482 | 0.561 | 0.662 |
|  |  |  | Time (day⁻¹) | 0.364 (-3.744 to 4.472) | 0.462 |  |  |
|  | Peak Power | 2 | Peak Power | 0.174 (-1.887 to 2.234) | 0.478 | 0.838 | 0.403 |
|  |  |  | Time (day⁻¹) | -0.145 (-0.962 to 0.672) | 0.266 |  |  |
| 2 | Mean Power | 1 | Mean Power | 0.015 (-0.467 to 0.497) | 0.941 | 0.453 | 0.270 |
|  |  |  | Time (day⁻¹) | -0.102 (-0.231 to 0.026) | 0.099 |  |  |
|  | Mean Power | 2 | Mean Power | 0.052 (-0.115 to 0.219) | 0.461 | 0.559 | 0.383 |
|  |  |  | Time (day⁻¹) | -0.104 (-0.220 to 0.012) | 0.068 |  |  |
|  | Peak Power | 1 | Peak Power | 0.062 (-0.294 to 0.419) | 0.683 | 0.468 | 0.291 |
|  |  |  | Time (day⁻¹) | -0.117 (-0.260 to 0.027) | 0.094 |  |  |
|  | Peak Power | 2 | Peak Power | 0.067 (-0.093 to 0.227) | 0.331 | 0.596 | 0.435 |
|  |  |  | Time (day⁻¹) | -0.127 (-0.267 to 0.012) | 0.066 |  |  |

Phase 3 (1–10 August, n = 3) was not estimated because it provided zero residual degrees of freedom; descriptive results only.
