# Supplementary material for: Case Report: Reduced lactate response despite maintained performance: a case study of a world-class Paralympic sprinter undergoing pre-games hypoxic training
Source: Front Sports Act Living. 2026 Sep 3;8:1910721. doi: 10.3389/fspor.2026.1910721 (PMC13582420; doi:10.3389/fspor.2026.1910721)
Supplement: Supplementary file 3 [file Table3.docx]

**Supplementary Table S3 |** Representative hypoxic repeated-/sprint-training studies and the present case. RSH, repeated-sprint training in hypoxia. Able-bodied RSH/SIH studies have reported increased maximal lactate, enhanced glycolytic markers, or improved repeated-sprint ability, whereas the present low-resistance HITH case showed reduced lactate at a maintained workload. A PubMed search (July 2026) identified no prior hypoxic-training intervention study in para-sprint athletes.

| **Study** | **Population** | **Modality** | **Hypoxic dose** | **Protocol** | **Key lactate / RSA outcome** |
| --- | --- | --- | --- | --- | --- |
| Faiss et al. 2013 (14) | 40 trained men (able-bodied) | Cycling RSH | ~3000 m | 8 sessions; 10-s sprints, 1:2 | ↑ sprints to exhaustion vs normoxia; ↑glycolytic/O₂-utilisation mRNA |
| Faiss et al. 2015 (15) | 17 XC skiers (able-bodied; upper-body) | Double-poling RSH | 3000 m (FiO₂ 13.8%) | 6 sessions; 4×5×10-s, 1:2 | ↑ repeated double-poling sprints; greater upper-limb perfusion |
| Puype et al. 2013 (9) | Recreational men (able-bodied) | Cycling sprint-interval in hypoxia | ~4500 m | 6-wk sprint-interval | ↑ glycolytic enzyme (PFK) activity |
| Trincat et al. 2016 (16) | 16 swimmers (able-bodied) | Swim RSH (hypoventilation) | Arterial desaturation | 6 sessions; 2×16×15 m | ↑ number of sprints; ↑[La]max |
| Brocherie et al. 2017 (8) | Meta-analysis, team-sport athletes | RSH (mixed) | Various | Meta-analysis | RSH improves sea-level repeated-sprint ability |
| Present case | T47 female 400-m para-sprinter (n=1) | Cycling HITH | ~3000 m (FiO₂ 14.5%) | 16 sessions; 2×6×25-s, low resistance | ↓ post-set lactate at maintained power; no previous para-sprint study identified |
