# Supplementary material for: Case Report: Reduced lactate response despite maintained performance: a case study of a world-class Paralympic sprinter undergoing pre-games hypoxic training
Source: Front Sports Act Living. 2026 Sep 3;8:1910721. doi: 10.3389/fspor.2026.1910721 (PMC13582420; doi:10.3389/fspor.2026.1910721)
Supplement: Supplementary file 4 [file Table4.docx]

**Supplementary Table S4 |** Session-by-session HITH details. Resistance was 0.025–0.03 kp·kg⁻¹ throughout; sprint duration was 25 s. Inter-sprint recovery was 50 s except in the final two sessions (5 and 10 August; 40 s). Power and blood lactate are shown for Set 1 / Set 2. † indicates a value that reached the analyzer's upper limit (“Hi”) and was recorded as 25.0 mmol·L⁻¹ (right-censored). — indicates no Set 2 (single-set session).

| **Date** | **Resistance (kp·kg⁻¹)** | **Sets** | **Sprint : recovery (s)** | **Mean power S1 / S2 (W)** | **Peak power S1 / S2 (W)** | **3-min lactate S1 / S2 (mmol·L⁻¹)** |
| --- | --- | --- | --- | --- | --- | --- |
| 8 May | 0.025–0.03 | 2 | 25 : 50 | 193 / 172 | 231 / 207 | 22.4 / 24.4 |
| 10 May | 0.025–0.03 | 2 | 25 : 50 | 202 / 176 | 239 / 201 | 18.4 / 21.9 |
| 29 May | 0.025–0.03 | 2 | 25 : 50 | 206 / 179 | 244 / 213 | 24.2 / 21.2 |
| 31 May | 0.025–0.03 | 2 | 25 : 50 | 207 / 180 | 246 / 206 | 19.8 / 20.9 |
| 3 Jun | 0.025–0.03 | 1 | 25 : 50 | 205 / — | 249 / — | 25.0† / — |
| 21 Jun | 0.025–0.03 | 2 | 25 : 50 | 214 / 183 | 257 / 221 | 25.0† / 23.0 |
| 24 Jun | 0.025–0.03 | 2 | 25 : 50 | 201 / 206 | 247 / 246 | 21.7 / 22.0 |
| 28 Jun | 0.025–0.03 | 2 | 25 : 50 | 209 / 210 | 258 / 244 | 22.1 / 23.6 |
| 1 Jul | 0.025–0.03 | 2 | 25 : 50 | 205 / 212 | 253 / 255 | 25.0† / 25.0† |
| 18 Jul | 0.025–0.03 | 2 | 25 : 50 | 205 / 210 | 250 / 256 | 23.1 / 22.7 |
| 22 Jul | 0.025–0.03 | 2 | 25 : 50 | 213 / 212 | 257 / 260 | 17.5 / 21.8 |
| 25 Jul | 0.025–0.03 | 2 | 25 : 50 | 215 / 213 | 270 / 261 | 22.9 / 20.0 |
| 29 Jul | 0.025–0.03 | 2 | 25 : 50 | 213 / 217 | 265 / 259 | 18.2 / 19.9 |
| 1 Aug | 0.025–0.03 | 2 | 25 : 50 | 215 / 218 | 265 / 260 | 17.7 / 17.8 |
| 5 Aug | 0.025–0.03 | 2 | 25 : 40 | 205 / 204 | 253 / 238 | 19.1 / 21.9 |
| 10 Aug | 0.025–0.03 | 2 | 25 : 40 | 204 / 210 | 251 / 244 | 17.2 / 19.8 |
